# WWC proteins as emerging biomarkers and therapeutic targets in cancer pathogenesis

**DOI:** 10.3389/fcell.2026.1712319

**Published:** 2026-02-05

**Authors:** Shuge Wang, Junzhe Li, Xianhua Xu, Shijie Xu

**Affiliations:** 1 Department of Pathology, Hainan Cancer Hospital, Affiliated Cancer Hospital of Hainan Medical University, Haikou, Hainan, China; 2 Department of Thoracic Surgery, Hainan Cancer Hospital, Affiliated Cancer Hospital of Hainan Medical University, Haikou, Hainan, China; 3 Medical Research Center, Hainan Cancer Hospital, Affiliated Cancer Hospital of Hainan Medical University, Haikou, Hainan, China; 4 Institute of Human Behavioral Medicine, Medical Research Center, Seoul National University, Seoul, Republic of Korea

**Keywords:** WWC, Hippo pathway, Wnt/β-catenin pathway, cancer, therapeutic targets

## Abstract

WW and C2 domain-containing (WWC) proteins have emerged as pivotal regulators of cancer initiation, progression, and therapeutic resistance. This review synthesizes current evidence demonstrating that WWC1, WWC2, and WWC3 exert context-dependent effects across malignancies, with WWC2 consistently functioning as a tumor suppressor while WWC1 and WWC3 display tissue-specific variability. By integrating and regulating multiple signaling pathways, particularly Hippo and Wnt, WWC proteins function as molecular scaffolds that regulate proliferation, apoptosis, epithelial-mesenchymal transition, and metastasis. This review provides a comprehensive overview of the molecular characteristics, biological functions, and clinical implications of WWC proteins, highlighting their potential as prognostic biomarkers and therapeutic targets for precision oncology.

## 1 Introduction

WW and C2 domain-containing (WWC) proteins represent an evolutionarily conserved family of scaffold proteins that coordinate diverse intracellular signaling pathways. These proteins are predominantly localized in the cytoplasm, particularly enriched in perinuclear regions, underscoring their roles in intracellular trafficking and the spatial regulation of signaling events (Sudol et al., 2001). In mammals, the WWC family comprises three closely related members: WWC1 (also termed KIBRA), WWC2, and WWC3, each exhibiting distinct but overlapping tissue distribution patterns (Kremerskothen et al., 2003; Yoshihama et al., 2012). WWC1 and WWC2 are abundantly expressed in the kidney and brain, with WWC2 also enriched in the testes (Höffken et al., 2023). WWC3 shows prominent expression in ovarian tissue, suggesting specialized functions in reproductive biology (Wennmann et al., 2014). Phylogenetic evidence indicates that WWC proteins originated from a common ancestral *WWC1*-like gene conserved among bilaterian organisms. Although mammalian genomes typically encode all three WWC members, evolutionary chromosomal rearrangements have led to the loss of the *WWC3* gene in mice, thereby limiting mouse models to *WWC1* and *WWC2* expression. These species-specific genomic alterations should be considered when interpreting data from mouse models (Nguyen et al., 2011; Wennmann et al., 2014). WWC proteins also display tightly regulated, tissue-specific expression patterns, suggesting critical roles in cellular differentiation and tissue homeostasis. Owing to their high structural homology, WWC proteins exhibit functional redundancy. This allows for compensatory interactions with diverse signaling molecules and coordinated regulation of multiple signaling cascades essential for maintaining cellular integrity and normal physiological function (Kremerskothen et al., 2003; Nguyen et al., 2011; Yoshihama et al., 2012).

**FIGURE 1:**
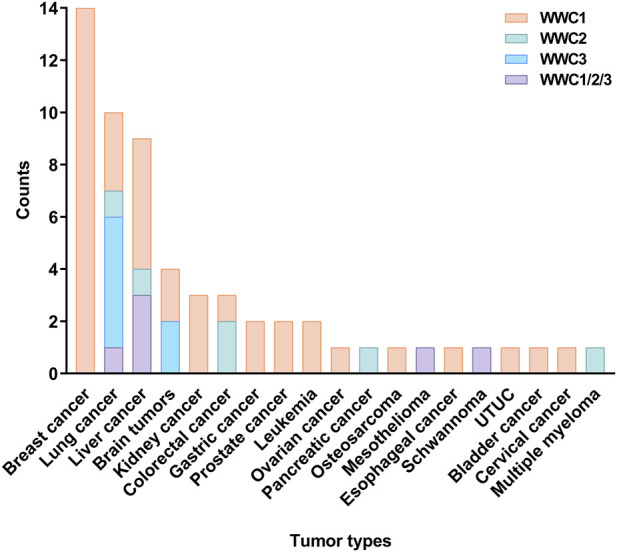
Distribution of publications on WW and C2 domain-containing (WWC) family genes across various tumors. The vertical axis represents the number of research papers, and the horizontal axis denotes the tumor types. Separate bars indicate the number of publications focusing on *WWC1, WWC2, WWC3,* and the combined total for all WWC genes for each tumor type. UTUC, upper tract urothelial carcinoma.

Emerging evidence suggests that WWC proteins are frequently dysregulated across a broad spectrum of human cancers. In most malignancies, their expression is significantly downregulated, correlating with tumor progression and poor clinical outcomes (Azimifar et al., 2014; Nagashima et al., 2023; Wang G. et al., 2020). Notably, *WWC2* is consistently downregulated across diverse cancer types, supporting its role as a tumor suppressor (Wang et al., 2022; Zhang et al., 2017). In contrast, *WWC1* and *WWC3* expression levels display greater variability and tissue-specific differences, reflecting distinct regulatory mechanisms and context-dependent functions in certain tumor types (Anuj et al., 2017; Huang et al., 2024; Wang Z. et al., 2019). Recent studies have underscored the involvement of WWC proteins in cancers of the brain, kidney, breast, lung, liver, and gastrointestinal tract (Figure 1). These proteins regulate tumor cell proliferation, migration, invasion, epithelial–mesenchymal transition (EMT), metastasis, and chemoresistance by modulating key signaling pathways, notably Hippo and Wnt/β-catenin. Consequently, WWC proteins are gaining recognition as promising biomarkers for prognostic evaluation and as potential therapeutic targets, including strategies aimed at overcoming drug resistance (Han et al., 2017; Hilton et al., 2008; Mudduwa et al., 2018; Ren et al., 2025; Wang S. et al., 2018; Wang Y. et al., 2018; Wang Z. et al., 2019). Despite growing evidence of their roles in cancer, the specific mechanisms, tissue-specific function, and clinical relevance of WWC proteins remain incompletely characterized, warranting a comprehensive review.

In this review, we comprehensively summarize current knowledge on the molecular structure, functions, regulation, and clinical significance of WWC proteins in cancer research. We also identify critical knowledge gaps and propose future research directions, emphasizing opportunities to advance precision oncology by targeting WWC-regulated signaling pathways.

## 2 Molecular structures and regulatory functions of WWC proteins

**FIGURE 2:**
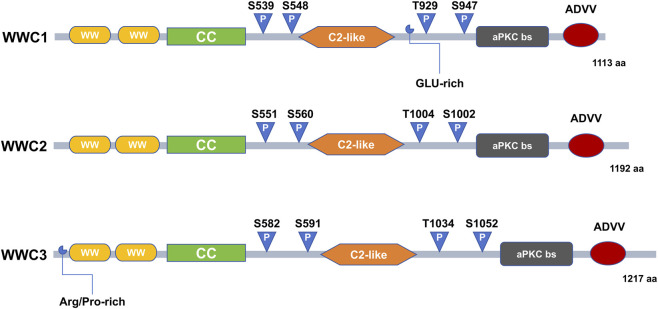
Domain structures of WWC1, WWC2, and WWC3 proteins. The schematic diagram illustrates the conserved and unique domains of the three paralogs of the WWC family. All three proteins WWC1 (1113 amino acids [aa]), WWC2 (1192 aa), and WWC3 (1217 aa) share two N-terminal WW domains, a coiled-coil domains (CC), an internal C2-like domain, and a C-terminal atypical protein kinase C (aPKC)–binding site (aPKC bs) and PDZ-binding motif (ADDV), reflecting a conserved core architecture. WWC1 contains a glutamic acid-rich region (GLU-rich), whereas WWC3 possesses a unique arginine/proline-rich region (Arg/Pro-rich). Multiple phosphorylation sites (P) are distributed throughout all WWC proteins.

WWC1 was originally identified through yeast two-hybrid screening as a scaffold protein encoded on human chromosome 5q34. The WWC family comprises three structurally related members: WWC1 (1113 amino acids [aa]), WWC2 (1192 aa), and WWC3 (1217 aa), which share highly conserved structural features (Figure 2). Each protein contains two WW domains in the N-terminal region (approximately residues 7–39 and 54–86), characterized by conserved tryptophan residues that bind proteins with proline-rich (PPxY) motifs. In addition, WWC proteins possess a conserved C2 domain consisting of two four-stranded β-sheets that mediate Ca^2+^-dependent phospholipid binding. The carboxy-terminal region includes an atypical protein kinase C (aPKC)–binding site and a conserved class III PDZ-binding motif (ADDV). Notably, WWC1 contains additional coiled-coil domains and glutamic acid-rich regions, whereas WWC3 has a distinctive N-terminal extension enriched in arginine and proline residues upstream of its WW domain (Kremerskothen et al., 2003; Wennmann et al., 2014). Studies suggest a functional redundancy among WWC proteins. For instance, WWC2 can partially compensate for WWC1 deficiency in mouse models, thereby preserving cellular homeostasis (Makuch et al., 2011).

WWC proteins modulate numerous cellular processes through complex interactions with critical signaling pathways. WWC1 functions primarily as a scaffold within multiprotein complexes that regulate cytoskeletal dynamics, thereby influencing cell migration, polarity, vesicular trafficking, and synaptic signal transduction (Duning et al., 2008; Kremerskothen et al., 2003; Rosse et al., 2009; Yoshihama et al., 2011). WWC2 plays essential regulatory roles in embryogenesis, angiogenesis, preimplantation embryonic lineage formation, oogenesis, and mitotic progression, thereby determining blastocyst cell fate (Hermann et al., 2021; Virnicchi et al., 2020). Similarly, WWC3 is critical for maintaining cellular homeostasis, particularly by inhibiting vascular remodeling via the Hippo signaling pathway (Chen and Liu, 2018) and by regulating the cell cycle, apoptosis, and cell adhesion (Yang et al., 2020). Increasing evidence indicates that dysregulated expression or functional impairment of WWC proteins contributes to pathological conditions, notably tumor initiation, malignant progression, and metastasis.

## 3 WWC proteins as key modulators of Hippo and Wnt signaling in cancer

WWC proteins act predominantly by modulating the Hippo signaling pathway, a highly conserved regulatory cascade that controls cell proliferation, apoptosis, tissue regeneration, and organ size (Yu and Guan, 2013). WWC1 is tightly regulated post-translationally, including by ubiquitin-dependent proteasomal degradation mediated via the Skip/Cullin/F-box^Slimb^ complex (Tokamov et al., 2021). Mechanical tension also influences the stability of the WWC1 protein, thereby affecting tissue growth and cellular dynamics. In cancer, WWC proteins primarily function as tumor suppressors by directly interacting with key components of the Hippo pathway, such as large tumor suppressor 1/2 (LATS1/2) kinases, mammalian Ste-20-like protein 1/2 (MST1/2) kinases, and the adaptor protein salvador homolog 1 (SAV1). Through these interactions, WWC proteins facilitate the assembly and stabilization of the SAV1–MST1/2–LATS1/2 kinase complex, enhancing MST1/2-mediated phosphorylation and subsequent activation of LATS1/2. Activated LATS1/2 then phosphorylates the transcriptional coactivator Yes-associated protein (YAP). Phosphorylated YAP is sequestered in the cytoplasm, preventing its nuclear translocation, which represses oncogenic transcription and inhibits tumor progression (Qi et al., 2022; Xiao et al., 2011).

In addition to regulating canonical Hippo signaling, WWC proteins modulate classical Wnt signaling. WWC proteins can bind essential components of the Wnt cascade, including glycogen synthase kinase 3 beta (GSK3β), Dishevelled 2 (Dvl2), and T-cell factor 4 (TCF4), thereby inhibiting β-catenin activation. By suppressing β-catenin–mediated transcriptional activity, WWC proteins effectively limit tumor cell proliferation, invasion, and metastasis (Han et al., 2017; Ren et al., 2025; Wang Y. et al., 2018).

**FIGURE 3:**
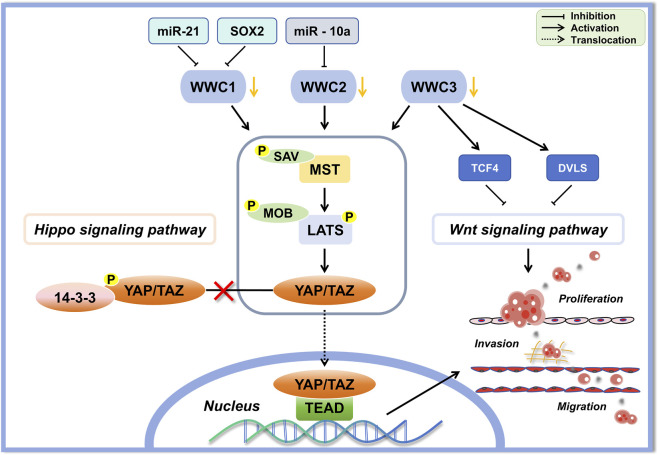
Schematic diagram depicting the regulatory roles of WWC proteins in the classical Hippo and Wnt signaling pathways in tumors. Reduction or inhibition of WWC proteins by microRNAs (miR-21, miR-10a) or transcription factors (SOX2) attenuates their inhibitory effect on the core Hippo kinase complex (comprising mammalian Ste-20-like protein 1/2 [MST1/2] and large tumor suppressor 1/2 [LATS1/2] kinases, the adaptor proteins MOB kinase activator 1 [MOB1] and salvador homolog 1 [SAV], and the transcription co-activators Yes-associated protein [YAP] and transcriptional co-activator with PDZ-binding motif [TAZ]), leading to YAP/TAZ dephosphorylation and nuclear translocation. Additionally, WWC proteins suppress Wnt signaling by interacting with key signaling components such as T-cell factor 4 (TCF4) and the Dishevelled (Dvl) family of proteins, thereby limiting tumor proliferation, migration, and invasion.

Furthermore, WWC proteins serve as integrative hubs for multiple signaling pathways through direct interactions and competitive binding with various upstream regulatory proteins. WWC proteins form homo- or heterodimeric complexes and bind proteins such as deleted in liver cancer 1 (DLC1), protein interacting with C kinase 1 (PICK1), aPKC, and LATS kinases, thereby coordinating processes such as remodeling of the cytoskeleton, establishment of cell polarity, and integration of diverse signaling events (Jin et al., 2015; Makuch et al., 2011; Rayala et al., 2006; Xiao et al., 2011). Collectively, these findings underscore the significance of WWC proteins as multifunctional regulators that influence tumorigenesis and cancer progression, primarily through the complex modulation of the Hippo and Wnt signaling networks (Figure 3).

## 4 The roles of WWC proteins in various cancers

### 4.1 WWC proteins in breast cancer

*WWC1* is a hormone-responsive gene expressed in breast epithelium that encodes a protein integrating hormonal signals and extracellular matrix interactions to maintain breast tissue homeostasis (Hilton et al., 2008). *WWC1* is frequently downregulated in breast cancer and is closely associated with advanced clinical stage, high histological grade, and negative estrogen receptor (ER) and progesterone receptor (PR) status. Furthermore, WWC1 expression levels inversely correlate with promoter DNA methylation, suggesting that epigenetic silencing may underlie its downregulation (Wang Z. et al., 2019). Low WWC1 expression is strongly associated with triple-negative breast cancer (ER−/PR−/HER2−) and with a lower Ki-67 proliferation index. It also correlates with poorer recurrence-free survival (RFS) in patients with luminal breast cancer receiving endocrine therapy or chemotherapy (Mudduwa et al., 2018). Additionally, genome-wide association studies have identified a significant relationship between the *WWC1* locus—specifically the region containing the single nucleotide polymorphism (SNP) rs147106204—and increased susceptibility to ER-positive breast cancer among African American women (Wang S. et al., 2018). However, conflicting data indicate that WWC1 is elevated in certain breast cancer cell lines and clinical specimens, suggesting that WWC1 may exert a potential tumor-promoting function in fibroblasts, possibly through repression of the tumor suppressor gene *RASSF1A* (Anuj et al., 2017). These contradictory observations may reflect differences in cellular context or tissue-specific regulatory mechanisms.

Mechanistically, loss of WWC1 expression reduces phosphorylation of LATS1/2 and YAP, thereby inducing EMT, while MST1/2 levels remain unaffected (Moleirinho et al., 2013). Conversely, increased WWC1 expression enhances Hippo pathway activity, effectively suppressing EMT and tumor progression (Zhang et al., 2016). Moreover, WWC1 loss promotes nuclear translocation of YAP1 and transcriptional co-activator with PDZ-binding motif (TAZ), which drives transcriptional activation of amphiregulin and subsequent epidermal growth factor receptor signaling, thereby sustaining tumor cell proliferation regardless of exogenous epidermal growth factor stimulation (Mussell et al., 2018). Furthermore, WWC1 is phosphorylated at multiple residues by the extracellular signal-regulated kinase–p90 ribosomal S6 kinase signaling axis, which critically regulates breast cancer cell proliferation and migration (Yang et al., 2014).

WWC1 also functionally interacts with critical regulators of breast cancer cell migration and invasion. Studies have shown that WWC1 directly binds to DLC1, an interaction that acts downstream of estrogen signaling to modulate ER-mediated transcriptional activation, thereby promoting cell proliferation (Rayala et al., 2006). Additionally, WWC1 forms a ternary complex with the discoidin domain receptor 1 (DDR1) tyrosine kinase and aPKCζ, which coordinates downstream signaling cascades essential for breast cancer metastasis (Hilton et al., 2008).

Collectively, these findings highlight *WWC1* as a tumor suppressor gene whose dysregulation significantly affects breast cancer progression, metastasis, and therapeutic resistance. However, *WWC2* and *WWC3* remain insufficiently studied in breast cancer, underscoring the need for comprehensive investigation of their specific roles and underlying mechanisms in breast tumorigenesis and progression.

### 4.2 WWC proteins in lung cancer

WWC proteins are generally downregulated in lung adenocarcinoma (LUAD). WWC1 expression in LUAD is negatively regulated by microRNA-21 (miR-21), which suppresses Hippo signaling, thereby promoting lung cancer cell proliferation and invasion while inhibiting apoptosis (An et al., 2018). Additionally, the oncogenic kinase thousand and one amino acid kinase 1 (TAOK1) directly interacts with WWC1, further reducing WWC1 levels. Notably, depletion of WWC1 expression reverses the tumor-suppressive effects of TAOK1 inhibition, highlighting a complex reciprocal regulatory interaction in LUAD (Chen, 2022). Similarly, WWC2 is frequently downregulated in LUAD, potentially through regulation by miR-21–5p (Wang G. et al., 2020). However, the precise molecular mechanisms by which WWC2 loss contributes to lung tumorigenesis remain to be fully elucidated.

WWC3 exhibits robust tumor-suppressive activity as a scaffold that regulates key signaling cascades in non-small-cell lung cancer (NSCLC). It suppresses tumor development through coordinated crosstalk between the Hippo and Wnt pathways. Specifically, the WW domain of WWC3 binds to the PY motifs of Dvl proteins, while its C-terminal ADDV domain engages the PDZ domains of Dvls. These interactions inhibit casein kinase 1ε-mediated phosphorylation of Dvl, thereby suppressing Wnt/β-catenin pathway activation. In addition, WWC3 promotes Hippo pathway activation through competitive binding with Dvl and LATS1. This dual modulation collectively inhibits proliferation, invasion, and metastasis in NSCLC cells (Han et al., 2017). Furthermore, WWC3 can directly activate the Hippo pathway by binding to LATS1 and enhancing its kinase activity, leading to inhibition of YAP nuclear translocation and downregulation of EMT-promoting factors such as Snail and Slug, thereby effectively suppressing EMT (Han et al., 2018).

Moreover, the function of WWC3 is modulated by multiple interacting partners. For example, the tumor suppressor FERM-domain-containing protein-1 (FRMPD1) binds the ADDV domain of WWC3 via its PDZ domain, thereby enhancing Hippo pathway activation (Rong et al., 2019). Conversely, the WW domain-binding protein 2 (WBP2) can competitively bind to the WW domain of WWC3 via its PPxY motif, competing with LATS1 and inhibiting Hippo pathway activation (Han et al., 2021). Furthermore, WWC3 suppresses starvation-induced autophagy through a Beclin1-independent mechanism, activating caspase-3/7 and promoting apoptosis. WWC3 expression levels negatively correlate with tumor cell proliferation and positively correlate with apoptosis, underscoring its critical role in regulating cell survival and death in lung cancer (Han et al., 2020).

In summary, the WWC family proteins (WWC1, WWC2, and particularly WWC3) function as critical tumor suppressors in lung cancer. By coordinated regulation of the Hippo and Wnt pathways, as well as autophagy, WWC proteins substantially influence tumor cell proliferation, migration, invasion, and EMT. Consequently, WWC proteins represent promising diagnostic biomarkers and therapeutic targets in lung cancer.

### 4.3 WWC proteins in liver cancer

WWC1 and WWC2 are broadly expressed in liver cell populations, including hepatocytes, Kupffer cells, sinusoidal endothelial cells, hepatic stellate cells, and bile duct cells (Azimifar et al., 2014). Consistently, both WWC1 and WWC2 expressions are significantly reduced in hepatocellular carcinoma (HCC) tissues, correlating with advanced tumor stage, poor clinical prognosis, and reduced patient survival (Hermann et al., 2018; Zhang et al., 2017). Functional studies have demonstrated that combined deletion of *WWC1* and *WWC2* in hepatocytes leads to dysregulated liver growth and tumor initiation. In contrast, individual knockout of either *WWC1* or *WWC2* exerts minimal effects on hepatocyte proliferation or liver size, reflecting functional redundancy between these proteins (Hermann et al., 2018).

In HCC, WWC proteins primarily act as tumor suppressors by activating the Hippo signaling pathway, thereby inhibiting EMT, invasion, and metastatic progression (Hermann et al., 2018; Zhang et al., 2017). Specifically, WWC1 collaborates with the tumor suppressor neurofibromatosis type 2 (NF2) to promote activation of LATS1/2 kinases, effectively repressing the oncogenic activity of the transcriptional coactivators YAP/TAZ (also known as WWTR1), thereby preventing the progression of intrahepatic cholangiocarcinoma (iCCA) (Park et al., 2020). Additionally, WWC proteins (WWC1/2/3), together with MST1/2 and SAV1, form a signaling module that robustly activates LATS1/2 and suppresses YAP/TAZ-mediated transcriptional programs (Qi et al., 2022). Consistent with this, a recently proposed “dual-module” model of Hippo signaling suggests two functionally distinct modules: the HPO1 (MST1/2–SAV1–WWC1–3–LATS1/2) and HPO2 (MAP4K1–7–NF2–LATS1/2) modules. Inactivation of either module leads to partial YAP/TAZ activation, promoting cholangiocyte proliferation and HCC development, whereas concurrent inactivation of both modules induces full YAP/TAZ activation and accelerates the progression of immature cholangiocytes into aggressive iCCA (Qi et al., 2023; Zhong et al., 2024; Zhong et al., 2023). Notably, the HPO1 module predominantly regulates organ size and exerts a stronger suppressive effect on YAP/TAZ compared to the HPO2 module.

In addition to canonical Hippo signaling via LATS activation, WWC proteins regulate this pathway through non-canonical mechanisms, particularly by stabilizing angiomotin (AMOT) proteins. Loss of WWC proteins destabilizes AMOT, enhancing YAP-driven transcription, and thereby promoting hepatocyte transdifferentiation, hepatic progenitor cell proliferation, inflammatory responses, fibrosis, and ultimately hepatocarcinogenesis (Hermann et al., 2018).

WWC proteins also modulate resistance to targeted therapies in liver cancer. For instance, microrchidia 2-induced hypermethylation of the NF2/WWC1 promoter suppresses the expression of these tumor suppressors, thereby activating YAP and enhancing the self-renewal of liver cancer stem cells, which ultimately contributes to therapeutic resistance and tumor progression (Wang T. et al., 2018). Additionally, the RNA-binding protein MEX3A downregulates WWC1, which inhibits Hippo signaling and promotes HCC cell proliferation, migration, and resistance to sorafenib treatment. Conversely, restoring WWC1 expression in combination with sorafenib markedly reduces tumor growth, highlighting WWC1 as a potential therapeutic target for overcoming drug resistance (Fang et al., 2023). Furthermore, WWC1 regulates the RhoGEF/RhoA/Hippo/CD44 signaling axis, which can effectively reverse regorafenib resistance in liver cancer (Zhou et al., 2024).

In summary, WWC proteins—particularly WWC1 and WWC2—function as key tumor suppressors in liver cancer by regulating the Hippo signaling pathway, suppressing oncogenic YAP/TAZ activity, and maintaining hepatic homeostasis. Their reduced expression is strongly associated with tumor progression, metastasis, and therapeutic resistance. These findings underscore their clinical significance as both prognostic biomarkers and promising therapeutic targets for effective treatment strategies in liver cancer.

### 4.4 WWC proteins in brain tumors

WWC proteins also play essential roles in normal neural function and in certain neurological disorders of the central nervous system. WWC1 has been implicated in core neural processes such as learning and memory (Ji et al., 2019; Mendoza et al., 2022; Papassotiropoulos and de Quervain, 2024), cognitive function (Li et al., 2020), and susceptibility to Alzheimer’s disease (Jurasova et al., 2024; Kauwe et al., 2024; Wang D. et al., 2019). WWC2 is predominantly localized at inhibitory postsynaptic densities, where it negatively regulates the surface expression of γ-aminobutyric acid type A (GABA_A_) receptors, thereby modulating inhibitory synaptic transmission (Dunham et al., 2024). However, owing to the evolutionary loss of WWC3 in mice, its specific role in mouse neural development and physiology remains unclear.

Although WWC proteins have been extensively studied in neuronal physiology, their functions in brain tumors—particularly gliomas—remain poorly understood. Gliomas are the most common primary brain malignancies, characterized by rapid growth, diffuse invasiveness, and poor prognosis (Weller et al., 2024; Sonoda, 2020). Notably, WWC1 expression is increases following inhibition of GSK3β-dependent Wnt signaling in glioma stem cells (Ren et al., 2025). This elevated WWC1 expression enhances LATS1 phosphorylation, thereby inhibiting the transcriptional activity of YAP. Consequently, glioma stem cell proliferation is reduced, c-Myc–driven oncogenic signaling is suppressed, and the tumors become more sensitive to chemotherapy (Ren et al., 2025).

WWC3 expression is significantly downregulated in gliomas, and the protein exerts potent tumor-suppressive effects. WWC3 directly binds to the transcription factor TCF4, thereby effectively inhibiting β-catenin–mediated Wnt signaling, which in turn suppresses glioma cell proliferation, migration, and invasiveness (Wang Y. et al., 2018). Moreover, WWC3 downregulation involves complex regulatory mechanisms mediated by the transcription factors BTB and CNC homology 2 (BACH2) and RNA-binding protein fused in sarcoma (FUS). Together, BACH2 and FUS relieve the repression of miR-10b-5p by the long non-coding RNA TSLNC8, resulting in persistently low WWC3 expression. Consequently, WWC3 downregulation attenuates Hippo pathway activity and promotes glioma progression (Yang et al., 2020).

Collectively, these findings highlight WWC proteins as crucial regulators of neural function and potent tumor suppressors in glioma pathogenesis, acting primarily through modulation of the Hippo and Wnt signaling pathways. Future research should aim to elucidate the molecular mechanisms by which WWC proteins influence glioma initiation and progression, thereby informing novel diagnostic and therapeutic strategies in clinical neuro-oncology.

### 4.5 WWC proteins in kidney cancer

WWC1 plays a pivotal role in maintaining renal architecture and function. It is highly expressed in glomerular podocytes, renal tubular epithelial cells, and collecting ducts. WWC1 mediates interactions between podocyte polarity complexes and cytoskeletal components, thereby preserving the structural integrity and functional stability of renal tissue (Duning et al., 2008). Both reduced and elevated WWC1 levels impair podocyte structure and function, highlighting the importance of balanced WWC1 expression for renal homeostasis (Lin et al., 2017; Meliambro et al., 2023).

In renal malignancies, particularly clear cell renal cell carcinoma (ccRCC), WWC1 expression is frequently and significantly decreased compared with normal kidney tissue. Low WWC1 levels correlate with higher tumor grade, advanced stage, larger tumor size, presence of distant metastasis, and poorer patient survival outcomes. Consequently, WWC1 is recognized as a potential prognostic biomarker and tumor suppressor in renal carcinogenesis (Nagashima et al., 2023). Mechanistically, WWC1 downregulation in ccRCC is associated with decreased LATS2 kinase activity and enhanced proliferative capacity of cancer cells. Moreover, epigenetic mechanism plays a critical role in regulating WWC1 expression. Hypermethylation of specific CpG islands in the WWC1 promoter, particularly at the CpG I site, leads to transcriptional silencing of WWC1. This hypermethylation directly represses WWC1 transcription and prevents the transcription factor SP1 from activating the WWC1 gene (Schelleckes et al., 2017). Notably, treatment with the demethylating agent 5-azacytidine reverses WWC1 promoter hypermethylation and restores WWC1 expression, suggesting that WWC1 is a promising epigenetically regulated therapeutic target.

In summary, WWC1 plays a dual role: maintaining normal renal tissue homeostasis and functioning as a critical tumor suppressor that inhibits ccRCC progression. The clinical significance of WWC1 downregulation in ccRCC underscores its potential as both a prognostic biomarker and a target for epigenetic therapeutic interventions.

### 4.6 WWC proteins in other malignancies

WWC family proteins have been implicated in numerous other solid tumors and hematologic malignancies, where they exhibit tissue-specific functions and engage in complex regulatory mechanisms during tumorigenesis and disease progression.

In colorectal cancer (CRC), the tumor-suppressor transcription factor TCF19 negatively regulates WWC1 expression, thereby promoting tumor progression (Du et al., 2020). Additionally, WWC2 expression in CRC is regulated by non-coding RNAs. The long non-coding RNA LINC00460 suppresses *WWC2* transcription by recruiting the ETS transcription factor ERG, whereas the circular RNA *circXPO1* promotes *WWC2* mRNA degradation by interacting with the RNA-binding protein FMRP. Together, these mechanisms suppress Hippo signaling, increase YAP activity, and promote EMT as well as tumor cell proliferation (Yuan et al., 2020; Zhu and Zhang, 2024).

In gastric cancer (GC), hypermethylation of the *WWC1* promoter significantly increases cancer susceptibility and, when combined with a high-salt diet, cooperatively enhances gastric carcinogenesis (Zhang et al., 2018). Notably, reduced aPKCλ expression facilitates elevated WWC1 levels to further suppress aPKC activity. This disruption of aPKCλ signaling in GC cells leads to loss of cell polarity, promotes lymphatic invasion, and is associated with poorer patient prognosis (Yoshihama et al., 2013).

In pancreatic cancer, microRNA-10a directly targets WWC2, thereby attenuating Hippo pathway activity, which elevates YAP-driven gene transcription, promotes EMT, and maintains cancer stemness (Wang C. et al., 2020). Similarly, in osteosarcoma, the transcription factor Sox2 directly represses WWC1 and NF2 expression, thereby activating YAP and maintaining stem cell–like features associated with tumor aggressiveness (Basu-Roy et al., 2015). In esophageal squamous cell carcinoma, SRY-related HMG box (SOX2) cooperates with ACTL6A and TP63 to repress WWC1 transcription, thereby enhancing invasive capacity and contributing to chemoresistance (Chai et al., 2019).

WWC2 is a key protein packaged into myeloma cell-derived exosomes. It activates the Hippo signaling pathway in fibroblasts from monoclonal gammopathy of undetermined significance (MGUS), thereby inducing *de novo* synthesis of miR-27b-3p and miR-214–3p. This, in turn, promotes fibroblast activation and the establishment of a tumor-promoting microenvironment, facilitating the progression of MGUS to multiple myeloma (MM) (Frassanito et al., 2019).

In diffuse pleural mesothelioma (DPM), WWC family proteins function within the HPO1 module of the Hippo signaling pathway to regulate YAP/TAZ activity. An engineered WWC1-derived construct, termed “SuperHippo,” robustly activates the HPO1 module and effectively suppresses tumor progression in preclinical models (Zhu et al., 2024). Moreover, NF2 loss in DPM leads to the compensatory accumulation of WWC1–3, which assemble into alternative HPO1 modules that partially limit tumor progression. In NF2-related schwannomatosis, concurrent deletion of NF2 and WWC1/2 rapidly induces aggressive schwannoma formation, emphasizing the critical role of WWC proteins in maintaining YAP signaling equilibrium (Wang et al., 2025).

In prostate cancer, WWC1 expression is upregulated and positively correlates with metastatic potential. Androgen receptor (AR) signaling promotes the assembly of a complex containing WWC1, aPKCζ, and the polarity protein Par3. This complex suppresses Hippo signaling, thereby facilitating tumor cell invasion and metastasis (Liu et al., 2022; Stauffer et al., 2016; Zhou et al., 2017). In contrast, in non-metastatic upper tract urothelial carcinoma, high WWC1 expression serves as an independent protective factor against tumor metastasis (Kurata et al., 2022). In bladder cancer, the WWC1 SNP rs755813 C allele is associated with reduced bladder cancer risk but does not significantly alter WWC1 expression levels, suggesting a post-transcriptional regulatory mechanism to exert its protective effect (Huang et al., 2020).

Among hematologic malignancies, WWC1 promoter hypermethylation occurs frequently in B-cell acute lymphoblastic leukemia, particularly in cases associated with ETV6/RUNX1 translocation (Hill et al., 2011). In chronic lymphocytic leukemia (CLL), decreased WWC1 expression correlates with adverse prognostic factors such as unmutated immunoglobulin heavy chain variable (IGHV) status and high CD38 expression, both indicative of poor clinical outcomes (Shinawi et al., 2012).

WWC3 expression is notably decreased in endometrial cancer associated with type 2 diabetes, potentially contributing to enhanced tumor invasiveness (Mujammami et al., 2022). In cervical cancer, tumor cell–derived extracellular vesicles transfer miR-146a-5p to recipient cell, which suppresses WWC2 expression, activates YAP signaling, and subsequently enhances actin cytoskeletal dynamics, thereby promoting cell migration and metastatic potential (Wang et al., 2022).

Collectively, WWC proteins exert profound regulatory effects on tumor initiation, progression, metastasis, and therapeutic resistance across diverse cancer types. By modulating the Hippo pathway and interconnected signaling networks, WWC proteins hold considerable promise as diagnostic biomarkers and as potential therapeutic targets in a wide range of malignancies.

## 5 Conclusion

Accumulating evidence indicates that WWC family proteins play critical, context-dependent roles in cancer initiation, progression, and metastasis. WWC proteins are frequently downregulated, consistent with their tumor-suppressive roles in most malignancies, including those of the breast, lung, bone, kidney, colorectum, brain, blood, liver, cervix, and pancreas. Conversely, the expressions of WWC proteins are elevated, suggesting context-dependent oncogenic functions in certain cancers, including gastric, prostate, upper tract urothelial and bladder cancers (Table 1). These contrasting patterns underscore the complex regulatory roles of WWC proteins, which are shaped by epigenetic modifications, protein–protein interactions, and involvement in multiple signaling pathways.

**TABLE 1:** Summary of reported alterations in WWC protein expression across various tumor types, their functional implications, and associated signaling pathways.

WWC	Cancer types	Level	Main function in cancer	Pathways	References
WWC1	Breast cancer	Low	Tumor suppressor; inhibits cell proliferation and migration (prevents EMT) in mammary epithelium; Associated with the claudin-low subtype breast cancer	Hippo; EGFR–MAPK	Hilton et al. (2008), Mudduwa et al. (2018), Mussell et al. (2018)
	Lung adenocarcinoma	Low	Tumor suppressor; inhibits proliferation, migration and metastasis (pro-apoptotic)	Hippo	An et al. (2018)
	Osteosarcoma	Low	Tumor suppressor; restricts cancer stemness and proliferation	Hippo	Basu-Roy et al. (2015)
	Renal cell carcinoma	Low	Tumor suppressor; loss of WWC1 associated with tumor development	Unknown	Nagashima et al. (2023)
	Colorectal cancer	Low	Tumor suppressor; inhibits proliferation and metastasis	Unknown	Du et al. (2020)
	Acute Lymphoblastic leukemia	Low	Tumor suppressor; loss of WWC1 associated with leukemia progression	Unknown	Hill et al. (2011)
	Chronic lymphocytic leukemia	Low	Tumor suppressor; loss of WWC1 associated with worse outcomes	Unknown	Shinawi et al. (2012)
	Gastric cancer	High	(i) The level of WWC1 promotor methylation is linked to higher GC risk, (ii) aberrant high WWC1 (with low aPKC) associates with increased invasiveness of GC cells	aPKC (polarity)	Yoshihama et al. (2013), Zhang et al. (2018)
	Prostate cancer	High	Tumor promotor; enhances tumor cell proliferation, migration and invasion (metastatic progression)	Hippo	Stauffer et al. (2016), Zhou et al. (2017)
	Upper tract urothelial carcinoma	High	Tumor promotor; promote invasion	Unknown	Kurata et al.(2022)
	Bladder cancer	High	Potential tumor modulator; WWC1 upregulation observed in tumors	Unknown	Huang et al. (2020)
WWC2	Lung adenocarcinoma	Low	Tumor suppressor; inhibits cell proliferation, migration and invasion	Unknown	Wang et al. (2020b)
	Hepatocellular carcinoma	Low	Tumor suppressor; inhibits EMT, invasion and metastasis	Hippo	Zhang et al. (2017)
	Cervical cancer	Low	Tumor suppressor; inhibits metastasis	Hippo	Wang et al. (2022)
	Colorectal cancer	Low	Tumor suppressor; inhibits migration, invasion and EMT	Hippo	Yuan et al. (2020), Zhu et al. (2024)
	Pancreatic cancer	Low	Tumor suppressor; inhibits EMT and cancer stem cell maintenance	Hippo	Wang et al. (2020a)
	Multiple myeloma	-	Tumor modulator; mediates tumor–stroma communication supporting tumor growth	miRNA signaling	Frassanito et al. (2019)
WWC3	Non-small cell lung cancer	Low	Tumor suppressor (context-dependent Wnt signaling modulation); inhibits cell proliferation, colony formation, invasion, and EMT; promotes apoptosis under stress	Hippo; Wnt; autophagy	Han et al. (2018), Han et al. (2017), Han et al. (2020), Rong et al. (2019)
	Glioma	Low	Tumor suppressor; inhibits cell proliferation and migration	Wnt; Hippo	Ren et al. (2025), Wang et al. (2018b), Yang et al. (2020)

WWC proteins exert their tumor-suppressive effects predominantly by modulating the Hippo signaling pathway, thereby regulating crucial cellular processes such as proliferation, apoptosis, migration, invasion, EMT, and maintenance of tissue homeostasis. By promoting activation of the core Hippo kinase cascade (MST1/2–SAV1–LATS1/2), WWC proteins enhance phosphorylation and cytoplasmic retention of YAP, which in turn represses oncogenic gene expression and tumor growth. In addition to Hippo signaling, WWC proteins also inhibit the canonical Wnt/β-catenin pathway, by interacting with Wnt signaling components to limit the transcriptional activity of β-catenin. By suppressing β-catenin–driven oncogenic programs, WWC proteins help limit tumor cell proliferation and invasiveness, as observed in gliomas and lung cancer. In addition, WWC proteins function as integrative signaling hubs by forming complexes with or competing against key regulators such as DLC1, PICK1, aPKC, AMOT, NF2, and LATS kinases, coordinating cytoskeletal dynamics, cell polarity, and signaling networks that collectively influence tumor progression and metastasis.

## 6 Future perspectives

Clinically, dysregulated WWC expression is closely associated with adverse prognostic features, including higher tumor grade, enhanced metastatic potential, therapy resistance, and reduced patient survival, underscoring the diagnostic and prognostic potential of WWC proteins. Therapeutic interventions that restore or augment WWC protein function have shown considerable promise. For example, in breast cancer, activation of MET signaling has been shown to upregulate WWC1 expression, thereby reactivating tumor-suppressive Hippo signaling, suggesting a feasible therapeutic approach (Liu et al., 2020). More broadly, targeting WWC-regulated pathways, including Hippo and Wnt signaling and their downstream effectors, offers a novel avenue to overcome drug resistance and suppress tumor progression across multiple cancer types.

Despite these advances, significant knowledge gaps remain, particularly regarding the specific roles and molecular mechanisms of WWC2 and WWC3 in diverse tumor contexts. Comprehensive functional studies are urgently required to clarify the distinct and overlapping contributions of individual WWC family members to cancer initiation, progression, and metastasis. Addressing these challenges through integrated molecular, genomic, and clinical approaches will deepen our understanding of WWC-mediated signaling networks and facilitate the development of more precise diagnostic tools and targeted therapeutic strategies, ultimately advancing precision oncology.
